# eHealth Literacy and Health Behaviors Affecting Modern College Students: A Pilot Study of Issues Identified by the American College Health Association

**DOI:** 10.2196/jmir.3100

**Published:** 2017-12-19

**Authors:** Rebecca Katherine Britt, William Bart Collins, Kari Wilson, Georgiann Linnemeier, Andrew Mark Englebert

**Affiliations:** ^1^ Department of Journalism and Mass Communication South Dakota State University Brookings, SD United States; ^2^ Brian Lamb School of Communication Purdue University West Lafayette, IN United States; ^3^ School of Communication Studies Indiana University South Bend South Bend, IN United States; ^4^ St Vincent Hospital Indianapolis Indianapolis, IN United States; ^5^ University of Madison Wisconsin Menasha, WI United States

**Keywords:** eHealth literacy, eHEALS, college student health, online health behaviors

## Abstract

**Background:**

The eHealth Literacy Scale (eHEALS) has been widely adopted by researchers to understand how eHealth literacy can be put into context. eHealth researchers need to know how to promote positive health behavior changes across college students, given the importance of the Internet to acquire and use health information. The American College Health Association identified a set of key health issues that affect college students today. By understanding how eHEALS might be related to college students’ maintenance of their health and their use of online health resources, researchers will be provided with a better understanding of eHealth literacy and its pragmatic implications for health campaigns and future interventions.

**Objective:**

The goal of the study was to examine what eHEALS reveals about college student health behaviors identified by the American College Health Association. To understand college student current health maintenance and their intentions to maintain their health and use online resources, the theory of planned behavior was used as the theoretical framework for the study.

**Methods:**

Data were collected via a survey of 422 college students that included the eHEALS measure and questions about health issues based on the recommendations of the American College Health Association. These questions asked about college student current health, subsequent use of online health resources, and their intention to maintain their health and make use of such resources in the future.

**Results:**

eHEALS was positively and significantly associated with all 8 areas of health issues identified by the American College Health Association for college student current maintenance of health and use of online health resources and for future intention of health maintenance and use of online resources. Key issues that emerged with eHealth literacy were maintaining safe sex practices and seeking out related information, seeking out information on an exercise regime, information on vaccinations, and maintaining a balanced diet.

**Conclusions:**

These results suggest several areas that may be targeted for future health campaigns toward college students. In addition, eHEALS was found to be a useful instrument for college students in the United States. Lastly, these results point to a need to deliver targeted information to college students, particularly since eHEALS captures literacy based on positively phrased items.

## Introduction

### Background

In recent years, Norman and Skinner [1] developed the eHealth Literacy Scale (eHEALS) to measure eHealth literacy, which refers to “the ability to seek, find, understand, and appraise health information from electronic sources and apply the knowledge gained to addressing or solving a health problem.” eHEALS has been widely adopted by researchers to understand how well eHealth actually works [2,3]. As a result, researchers have found that as eHealth literacy rises, so does the ability to use online health resources effectively [4]. Factors that tend to predict how individuals behave, such as the use of online health resources, include their current use of health resources, past and future intent to use those resources, and self-maintenance of their own health. These factors are ones explained by Ajzen's theory of planned behavior (TPB) [5], which suggests that human action is guided by belief and motivation.

Despite its accolades, eHEALS has only begun to be explored with college students, who are regularly exposed to propaganda and Internet media on health issues and face a number of health issues such as social pressures, maintaining a healthy diet, getting enough sleep, and living with stress from balancing classes, relationships, and work [6]. eHealth literacy can have significant consequences on the quality of health information sought and retained [1,7-10]. Many college students remain uninformed about these issues as well as others identified by the American College Health Association (ACHA) [9-12]. In particular, electronic health literacy remains a vital issue to address among college students, not only so that we can understand their literacy, but so we can create appropriate interventions.

The objective of this study was to address eHEALS and its association with college student health behaviors based on past, current, and future behaviors [5]. In this study, we targeted the 8 areas that the ACHA [12] determined were critical health issues for college students by surveying students at a range of institutions across the United States. eHEALS has already received accolades for its efficacious assessment of eHealth literacy and has been used and adopted across cultures [13] and contexts [7,14-15]. We examined the relationship between eHEALS and intention to engage in healthy practices based on the ACHA’s 8 recommended areas. An individual’s intent, past behavior, future intent to engage in the use of online health resources, and self-maintenance of one’s health are all explained by key determinants of TPB [5]. TPB can indicate how much individuals are willing to care for their health or spend time seeking out online health resources. With eHEALS, a scale that addresses eHealth literacy on a broad level, we can better understand these behaviors.

### Theory of Planned Behavior

TPB was developed by psychologist Icek Ajzen [5,16], and according to TPB, human action is guided by 3 kinds of considerations: beliefs about the likely outcomes of the behavior and the evaluations of these outcomes, beliefs about the normative expectations of others and motivation to comply with these expectations, and beliefs about the presence of factors that may facilitate or impede performance of the behavior [5,16]. These are respectively known as behavioral intent, subjective norms, and perceived behavioral control. Central to TPB is the intention to perform a given behavior. Intentions are assumed to capture the motivational factors that influence a behavior; they are indications of how hard people are willing to try, of how much of an effort they are planning to exert in order to perform the behavior [5].

TPB has been used in various health contexts to explain an individual’s intent to engage in some future behavior, such as diet and fasting [17], obtaining Pap smears [18], mentally adjusting after diagnosis of cancer [19], smoking [20], and other issues. In addition, TPB has been found to be a predictor of future intention to engage in safe sex practices by college students [21], drinking behavior [22], and exercise [23]. TPB has not been examined in the context of electronic health literacy using eHEALS, but the past success of eHEALS [2,3,13,24] and TPB [17-22] research suggest a possible relationship. Health literacy naturally includes recognition of the broader domain of health behaviors—literacy implies an understanding of the context surrounding a particular health care decision. This includes knowledge of others’ health behaviors and choices in similar situations—the normative behaviors that guide one’s decisions under TPB. In other words, eHealth literacy is partially dependent upon the opportunities and behaviors that others follow throughout their health care, a construct that also guides TPB.

### Electronic Health Literacy

eHealth resources allow patients, providers, consumers, and caregivers to make better health-based decisions [9]. Health literacy is, as defined in the US Department of Health and Human Services’ *Healthy People 2010* report, “the degree to which individuals have the capacity to obtain, process, and understand basic health information and services needed to make appropriate health decisions” [25]. As Norman and Skinner [1] define it, eHealth literacy is the actual ability to seek, find, and make use of online health information. eHealth literacy goes beyond basic reading ability by understanding and synthesizing online health information to make informed choices and increase overall quality of life [26]. eHealth literacy is multifaceted and requires the skills of basic literacy, basic health literacy, and actual retrieval of information. Our study uses Norman and Skinner’s [1] definition since it closely matches the goals of this research.

### eHealth Literacy Scale

eHEALS is widely used today by researchers to measure eHealth literacy [2,13,27,28]. eHEALS is an 8-item measure of eHealth literacy designed to measure an individual’s knowledge and ability to find electronic health information and apply that information to health issues. The scale uses a 5-point Likert scale to rate the statements “I know what health resources are available on the Internet,” “I know how to use the health information I find on the Internet to help me,” “I know how to find helpful resources on the Internet,” “I have the skills I need to evaluate the health resources I find on the Internet,” “I know how to use the Internet to answer questions about my health,” “I know where to find helpful resources on the Internet,” “I can tell high-quality health resources from low-quality health resources on the Internet,” and finally, “I feel confident in using the information from the Internet to make health decisions.” Factor loadings for the original scale ranged from .60-.84 among 8 items.

eHEALS is one of the earliest scale developments to address a need for eHealth literacy for a wide population. Current research has retested eHEALS, although scholars have stated that there is a continued need to do so [7]. eHEALS has been shown to be a reliable and easy-to-use scale. It is based on 6 types of literacy skills: reading, health, information, scientific, computer, and media literacy. In terms of its widespread use, eHEALS has been translated to German [27], Japanese [13], and Dutch [7]. eHEALS tends to be used with specific health issues such as colorectal cancer in Japan [13], where it was found to be positively associated with cancer knowledge. Individuals scoring higher on eHEALS were more likely to undergo cancer screenings [13]. eHEALS also tends to be correlate with finding credible Internet sources; for instance, previous exposure to credible online health resources tends to be associated with higher levels of health literacy [28]. More recently, eHEALS scores have been associated with the digital divide among low-income older adults who had a depression diagnosis [2]. Recent scholarship has suggested that as the landscape of the Internet evolves, so do measures of ehealth literacy, including eHEALS; nonetheless, it represents a clear measure of literacy [29].

With growing choices that students can make about their health today—such as getting vaccinated for the human papillomavirus, provided at some universities, or how to balance school and increasing work demands—there is little wonder that many turn to the Internet to seek out information [30,31]. There have yet to be studies published that use eHEALS to better understand college students’ motivation, beliefs, and behaviors associated with online health resources and issues that are most salient to them.

### eHealth Literacy Scale and Broader Health Issues Pertinent to College Students

eHEALS was used to seek out the relationship between the scale and behaviors across a range of health issues identified by the ACHA. Emerging research has explored eHEALS to examine college student beliefs and behaviors relevant to the health issues that tend to affect them the most [32], the potential of eHEALS as a reliable and consistent measure that captures eHealth literacy [1,7], and more broadly, in developing health information technologies [33]. In addition, scholarship has noted an increased need to include theoretical frameworks to assist in developing, tailoring and executing online health research [24,34].

The current executive summary from the ACHA reports that the 8 most common indexes for college student health include drug use, sleep, sexual health, getting vaccinations, proper diet, maintaining friendships, maintaining an exercise regime, and overall general maintenance of health [12]. Regarding the term of general health, there is no additional work on its inclusions/exclusions, and as such, we treated it as a broad index. Delivering information online has become a necessity as most college students report using the Internet to retrieve information about health and well-being. In a 2009 survey by the Pew Research Center’s Internet and American Life Project, 56% of adults reported accessing the Internet, and 80% of Internet users have sought out health information online. Seeking out health information is the third most popular pursuit tracked by the Pew Research Center [35]. Given this, we propose the following hypotheses:

H1: eHEALS will be significantly related to college students’ general health, exercise regime, sleep, getting vaccinations, and maintenance of sexual health, a balanced diet, stable friendships, and a lifestyle free of harmful substances.

Electronic health literacy focuses on individual capacity to use electronic resources appropriately and as such, we would expect individual patterns of health information-seeking to be related to their overall level of electronic health literacy. Therefore,

H2: eHEALS will be significantly related to college students’ current use of Internet health resources in the areas of general health, exercise regime, sleep, getting vaccinations, and maintenance of sexual health, a balanced diet, stable friendships, and a lifestyle free of harmful substances.

Finally, we expect that electronic health literacy will be related to intention to maintain a healthy regime as well as intention to seek out additional Internet resources in these areas. Therefore,

H3: eHEALS will be significantly related to college students’ future intention to maintain a healthy regime in the areas of general health, exercise regime, sleep, getting vaccinations, and maintenance of sexual health, a balanced diet, stable friendships, and a lifestyle free of harmful substances.

H4: eHEALS will be significantly related to college students’ future intention to seek out Internet sources in the areas of general health, exercise regime, sleep, getting vaccinations, and maintenance of sexual health, a balanced diet, stable friendships, and a lifestyle free of harmful substances.

## Methods

### Overview

Following institutional review board approval, this study was conducted among a population of college students at a large midwestern university in the United States. An online survey was developed that included the eHEALS measurement [1] along with a series of planned behavior items to assess college students’ intention to manage their health [5,36]. Recruitment took place via a liberal arts online recruitment system, where students had the option to take the survey in exchange for a small amount of extra credit in a course of their choice. Students had the option to opt out of the study for an alternative extra credit activity.

Participants took a survey that was created to assess students’ use of the Internet to address health concerns or issues. A total of 420 participants participated in the study, ranging in ages from 18 to 35 (mean 20.48, SD 2.14) years, and the majority of participants were undergraduate students (mean 2.76, SD 1.15). Participants reported race/ethnicity of white (330/420, 78.6%), Asian/Pacific Islander (48/420, 11.4%), African American (15/420, 3.8%), Hispanic/Latino (14/420, 3.3%), other (11/420, 2.6%), and 4 missing values.

Participants first answered a series of demographic questions, followed by a question that asked whether or not they have a health condition that requires regular interaction with a physician. Participants were not asked to elaborate on this answer, and no participant chose to elaborate. The majority of participants (380/420, 90.6%) reported not having a major health condition, but 9.4% (39/420) did, with 3 missing values. To understand the level of past behavior, current behavior, and intent to participate in future behaviors, a series of questions were asked relating to each of the major dimensions of health items as identified by the ACHA [12]: general health, exercise, substance abuse, sleep, vaccination, sexual health, diet, mental health, and maintaining friendships. Upon completion of those questions, participants clicked a link to log their answers in the system and were thanked for their time.

### Measures

In the online survey, items provided measures of intent, attitudes, subjective norms, perceived behavioral control, and eHEALS. To answer the hypotheses, correlations were used as these best answered the questions at hand [36-37].

### Electronic Health Literacy

Electronic health literacy was assessed through the 8-item eHEALS measure through the average of all items measured on a 5-point Likert-scale (mean 3.99, SD .71). The eHEALS items were designed to solicit self-report assessments of knowledge of or comfort in finding, evaluating, or using Internet-based health information resources (eg, “I have the skills I need to evaluate the health resources I find on the Internet” and “I feel confident in using the information from the Internet to make health decisions”). The scale evidenced high internal consistency (Cronbach alpha=.897).

### Behavior

Behavior was measured with a single item for each of the 8 health areas identified from the ACHA, for example: “I have maintained a balanced sleep schedule (approximately 7 to 8 hours per night) so far this semester.” The phrasing of the behavior items was taken directly from Ajzen’s [36] recommended phrasing for TPB questions. Table 1 shows the descriptive statistics for each area of healthy maintenance.

### Intentions

Following typical recommendations for TPB research [5,16], we measured intentions toward using the Internet for each of the 8 areas identified from the ACHA (See Table 2). The phrasing of the intention items was taken directly from Ajzen’s [36] recommended phrasing for TPB questions. Measuring intent allowed us to assess a baseline of mindfulness for using the Internet for each of these identified issues.

**Table 1:** Descriptive statistics for self-reported past behavior for 8 areas of health issues.

Participant self-report of past behavior	Mean (SD^a^)
Overall health	3.98 (0.786)
Exercise regime (at least 2.5 hours per week)	3.33 (1.29)
Maintain lifestyle free of harmful substances	3.32 (1.32)
Sleep (approximately 7 to 8 hours per night)	2.91 (1.20)
Get necessary vaccinations	3.68 (1.16)
Maintain safe sex practices	4.18 (0.917)
Maintain balanced diet	3.48 (1.00)
Maintain positive social relationships	4.41 (0.676)

^a^SD: standard deviation.

**Table 2:** Descriptive statistics for self-reported intention items for the 8 areas of health issues.

Participant self-report of intention to use the Internet	Mean (SD^a^)
Overall health	3.30 (1.01)
Exercise regime (at least 2.5 hours per week)	3.96 (1.17)
Maintain lifestyle free of harmful substances	3.64 (0.109)
Sleep (approximately 7 to 8 hours per night)	3.28 (1.12)
Get necessary vaccinations	3.68 (1.12)
Maintain safe sex practices	3.90 (0.190)
Maintain balanced diet	4.08 (1.13)
Maintain positive social relationships	4.44 (0.234)

^a^SD: standard deviation.

## Results

**Figure 1:**
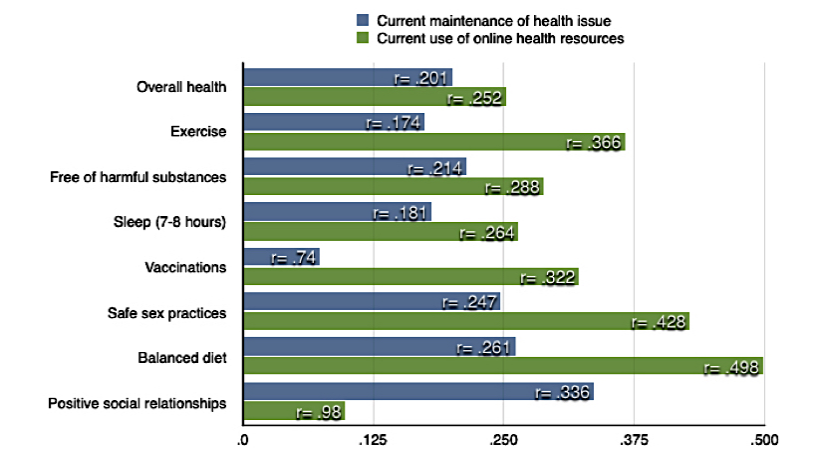
eHealth Literacy Scale correlated with current maintenance and current use of online health resources.

The first hypothesis predicted that electronic health literacy would be significantly correlated with an individual’s general health, exercise regime, sleep, getting vaccinations, and maintenance of sexual health, a balanced diet, stable friendships, and a lifestyle free of harmful substances. The results of the survey support this as shown in Figure 1.

Because our researchers come from a communication background in the behavioral sciences, correlation interpretation guidelines were based on the typical guidelines of Cohen [38], who argues that *r*=.100 corresponds to a small relationship; *r*=.243 and above is a moderate relationship, and *r*=.371 and above is a large relationship. Other behavioral scholars, such as Losh [39], have cited *r*=.01 to *r*=.25 as a weak relationship, where *r*=.26 to *r*=.50 is moderate, and *r*=.51 to *r*=.75 is strong. The *P*<.01 standard was used for all analyses.

In addressing the first hypothesis, eHEALS was positively and significantly correlated with all 8 areas of health identified by the ACHA. Most notably, self-report of an individual’s current maintenance of positive social relationships (*r*=.336, *P*=.001), a balanced diet (*r*=.261, *P*=.001), and practicing safe sex (*r*=.247, *P*=.001) emerged.

The second hypothesis predicted that eHEALS would be significantly related to college student current use of Internet health resources in the areas of general health, exercise regime, sleep, getting vaccinations, and maintenance of sexual health, a balanced diet, stable friendships, and a lifestyle free of harmful substances. Out of the 8 areas of health, 7 were significant at the *P*<.01 level; the exception was maintaining positive social friendships (*r*=.098, *P*=.05). 

Current use of online health resources approached a moderate relationship for seeking out information in the following areas: a balanced diet (*r*=.498, *P*=.001), safe sex practices (*r*=.428, *P*=.001), exercise (*r*=.366, *P*=.001), and vaccinations (*r*=.322, *P*=.001). Sleep was significantly and negatively correlated with use of online health resources (*r*=–.264, *P*=.001).

Hypothesis 3 predicted that future intention to maintain health would be related to eHEALS. Maintaining positive social relationships (*r*=.456, *P*<.01), balanced diet (*r*=.358, *P*<.01), and safe sex practices (*r*=.332, *P*<.01) were among the highest correlations addressing this question. Although all other areas emerged as significant and positive correlations, relationships were much smaller.

Finally, the fourth hypothesis predicted that future intention to use online health resources would be significantly related to eHEALS. The strongest relationships were found in this area, with several variables approaching a moderate correlation. Among these, diet (*r*=.486, *P*=.01), the intention to maintain general health (*r*=.451, *P*=.001), exercise (*r*=.380, *P*=.001), intending to maintain safe sex practices (*r*=.378, *P*=.001), maintaining a lifestyle free of harmful substances (*r*=.333, *P*=.001) and getting vaccinations (*r*=.332, *P*=.001) were the strongest relationships. The two weakest relationships included sleep (*r*=.213, *P*=.02) and maintaining positive social relationships (*r*=.187, *P*=.02).

## Discussion

### Principal Findings

In this study, we sought out what eHEALS reveals about college student health behaviors, focusing on health issues recommended by the ACHA. With numerous online options available for information-seeking behaviors, it is important to intimately know the audience and the issues that can inform campaign design and evaluation [40].

We learned that maintaining a balanced diet remains an important issue for college students, particularly as campus food [41-42] options increase in variety and more types of food choices become available to students.

In particular, online health campaigns can be designed for specific college campuses that employ the use of technologies. For example, previous research has evaluated the design, usability, and acceptability of social media resources for chronic health conditions [43] that reinforce the need to use eHEALS as a measure prior to campaign design. Previous campaigns have suggested that some implementation problems lie in understanding the target audience and their actual needs and literacy level [40-42]. We suggest that development of online campaigns geared toward college students should make use of eHEALS as an evaluative measure via pre/post-test; this can be critical in addressing areas of diet and health, especially if they are tailored to students at various institutions.

Next, it is important for researchers to use theory to help create online interventions for college students that use eHEALS in a theory-based context. For example, TPB allowed us to gain a better sense of student intentions to make use of online resources. Results that were consistent in all findings were maintenance of safe sex practices, diet, and positive social relationships. Internet campaigns have an opportunity to reach student populations, particularly as scholarship in JMIR has noted the need to support the effective use of technology for students [43]. More specifically, as researchers develop tailored Internet campaigns toward specific issues [44], a theory-based intervention can be a useful framework to help gauge attitudes toward health issues and intent to actually engage with a healthy behavior.

### Limitations

This study had several limitations. First, recruitment occurred through a single university. In order for these results to be more generalizable, researchers at different institutions should examine eHEALS among college students; for instance, rurally located universities in medically underserved areas would benefit from similar studies. In addition, tailored interventions for different colleges require different expectations and carefully crafted messages and multimedia design use, especially since Internet-based campaigns may not be the best mechanism for all institutions. Lastly, it is important to note that in this study, participants were recruited through a liberal arts recruitment system. While participants came from a broad range of academic backgrounds, recruitment from different universities entirely would be helpful in better understanding eHealth literacy, critical health issues for college students, and addressing those in the best manner possible.

### Conclusions

This study focused on the relationship between eHealth literacy and health issues that are crucial for many college students. Sexual health emerged as a primary concern, along with diet and maintaining vaccinations. This means that education about safe sex practices are key areas for researchers to target, and the use of online interventions can mitigate possible barriers and unintended effects of traditional face-to-face and mass media campaigns [45]. Developing online interventions, particularly for sensitive issues that relate to sexual health for young adults, will continue to be important in colleges and universities. Knowledge of how eHEALS helps us understand this group is particularly helpful in spurring these efforts. As a result of understanding how eHEALS works with TPB variables, we can begin to see how eHealth literacy is critical to study in an age where we are faced with myriad communication technologies.

## Abbreviations

ACHA: American College Health Association
eHEALS: eHealth Literacy Scale
TPB: theory of planned behavior
